# An instrument design for non-contact detection of biomolecules and minerals on Mars using fluorescence

**DOI:** 10.1186/1754-1611-8-16

**Published:** 2014-07-01

**Authors:** Heather D Smith, Christopher P McKay, Andrew G Duncan, Ronald C Sims, Anne J Anderson, Paul R Grossl

**Affiliations:** 1 Department of Biological Engineering, Utah State University, Logan, UT, USA; 2 NASA Ames Research Center, Space Science Division, Moffett Field, CA, USA; 3 Desert Sensors, Logan, UT 84341, USA; 4 Department of Biology, Utah State University, Logan, UT, USA; 5 Department of Plants, Soils and Climate, Utah State University, Logan, UT, USA

## Abstract

We discuss fluorescence as a method to detect polycyclic aromatic hydrocarbons and other organic molecules, as well as minerals on the surface of Mars. We present an instrument design that is adapted from the ChemCam instrument which is currently on the Mars Science Lander Rover *Curiosity* and thus most of the primary components are currently flight qualified for Mars surface operations, significantly reducing development costs. The major change compared to ChemCam is the frequency multipliers of the 1064 nm laser to wavelengths suitable for fluorescence excitation (266 nm, 355 nm, and 532 nm). We present fluorescence spectrum for a variety of organics and minerals relevant to the surface of Mars. Preliminary results show minerals already known on Mars, such as perchlorate, fluoresce strongest when excited by 355 nm. Also we demonstrate that polycyclic aromatic hydrocarbons, such as those present in Martian meteorites, are highly fluorescent at wavelengths in the ultraviolet (266 nm, 355 nm), but not as much in the visible (532 nm). We conclude that fluorescence can be an important method for Mars applications and standoff detection of organics and minerals. The instrument approach described in this paper builds on existing hardware and offers high scientific return for minimal cost for future missions.

## Introduction

A practical difficulty with organic and biological analysis on Mars missions is getting to, and collecting, the sample. Rovers remotely operated from Earth can take many days to drive to a site and to collect a sample. For this reason there is considerable interest in selection of target samples – both rock and dirt - from a distance of several meters.

The current method for non-contact detection on the *Mars Science Laboratory* (MSL) is *ChemCam. ChemCam* employs Laser-Induced Breakdown Spectrometer (LIBS) and can accomplish elemental chemical determination [1]. ChemCam consists of two instruments: 1) a remote micro-imager (RMI) capable of mm resolution from meters away and 2) a laser-induced breakdown spectrograph (LIBS) capable of determining certain elemental concentrations as low as 10 ppm [1]. The ChemCam instrument sits on the *Curiosity* rover mast 1.8 meters above the ground allowing for remote analysis at distances up to 9 meters [1]. The ChemCam instrumentation has achieved several technical breakthroughs including the first flight-qualified laser. All of the ChemCam hardware, including the excitation and emission systems, have achieved flight qualification, and are at Technical Readiness Level (TRL) 9 now that MSL is operating on the martian surface. A major addition in standoff detection would be the ability to detect low levels (ppm and less) of organics in rock and soil samples. Two methods are under consideration for this task: Raman spectroscopy and UV fluorescence. Standoff detection of organics by both Raman [2,3] and fluorescence [4] have been demonstrated in laboratory trials. In general, Raman spectroscopy provides better identification of organics than fluorescence while fluorescence provides a higher sensitivity to low levels of organics. In this paper we focus on fluorescence as a method to do stand-off detection of biological, organic, and mineralogical assays on future Mars missions.

Characterization of targets via fluorescence involves both an excitation wavelength and an emission wavelength. The excitation that results in fluorescence occurs when a photon of the appropriate wavelength reaches, or excites, the target and the energy state of an electron is raised. As the energy dissipates, the electron cascades down to lower energy states. The energy released in this cascade is emitted as photons of wavelengths longer than the excitation wavelength due to the loss of energy required to raise the electron state.

Previous investigations have employed the use of native fluorescence for mineral identification, organics, and photosynthetic compounds for over 100 years [5-16]. One such investigation examined using native fluorescence spectra of chlorophyll a and other photosynthetic pigments in their natural environment using airborne assets was first reported by Hoge and Swift [5]. A more extensive investigation using various pigment-protein Macromolecules in the 480–560 spectral region demonstrated the capability of measuring a variety of photosynthetic pigments using native fluorescence [6]. More recent investigations measured the fluorescence of several photosynthetic pigments to determine the microbial community of photosynthetic organisms [15,16]. A few in-situ fluorescence instruments have been developed to identify biosignatures and are at various stages of flight technical readiness levels. These instruments are aimed at detecting microbes by adding a fluorescing reagent [17] or within a fairly uniform, low fluorescence material such as ice [18,19] and in the ocean [20]. Work has begun to look at native fluorescence in soil as well [21]. These instruments provide a fluorescent method for detecting biomolecules and chemical organics.

In addition to detecting biomolecules and chemical organics, fluorescence can also be used as a tool for mineralogical identification [22] to aid in target selection. Many minerals are fluorescent when excited at certain excitation and emission wavelength combinations, for example, the mineral fluorite derives its name after this characteristic fluorescence property. Often in planetary applications spectral information requires information on the geological context before a mineral is identified. This does not diminish the utility of spectral data. Mineral and rock species identified on the surface of Mars include hematite, jarosite, olivine, phylosilicates, carbonates, perchlorate (ClO_4_), basalts, and ice among others [23,24].

### Modified ChemCam Instrument

The LIBS system that forms the core of ChemCam is a suitable starting point for future combined LIBS/Raman or LIBS/fluorescence instruments. Laser induced breakdown spectroscopy operates by heating/energizing the target surface producing a plasma. Elements are identified and their concentration determined based on the strength of atomic emission lines in the spectrum. ChemCam uses a 5 ns pulsed 1064 nm laser with a 15 hz firing rate drawing 30 mJ per pulse to produce the plasma [1]. To capture the emission spectrum of the plasma, ChemCam has a Schmidt telescope to increase the light gathering power before feeding the signal into three spectrometers. The spectrometers are customized versions of off-the-shelf Ocean Optics HR2000 spectrometers, each designed to measure specific wavelengths of the emission spectra (240–336 nm, 380–470 nm, 500–800 nm respectively). In a typical application the signal is integrated over 75 laser pulses. The combined spectrometer detection ranges from ultra-violet (UV) 240 nm to red 800 nm [1].

**Figure 1:**
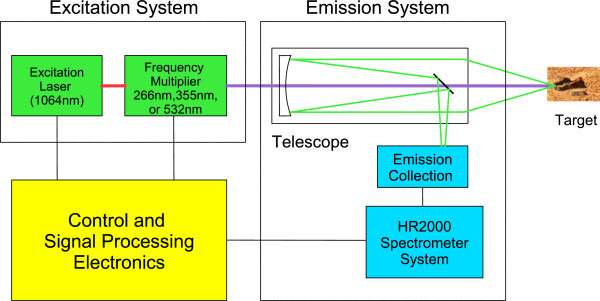
**System level block diagram of the modified ChemCam instrument.** Frequency multipliers are added to the excitation system for optimization of biomolecular, organic, and mineral flourescence detection. Diagram adapted from [1].

**Figure 2:**
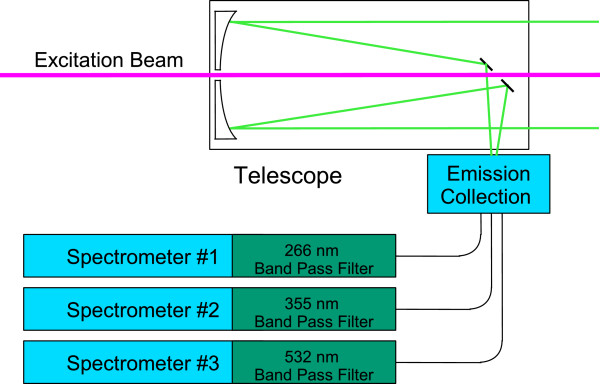
Emission Collection System enabling biomolecule, organics, and minerals detection by specific wavelength flourescence emission showing the appropriate filter blocks.

The primary component of the ChemCam instrument that enables fluorescence assessment is the 1064 nm laser. This wavelength (1064 nm) is too long of a wavelength, hence too low–energy to induce fluorescence. However with the aid of a frequency multiplier, irradiation can be produced at the three harmonic wavelengths, double, triple, and quadruple of the frequency of the original 1064 nm light, corresponding to wavelengths of 532 nm, 355 nm, and 266 nm, respectively. Biological material, organics, and minerals fluoresce when excited at 266 nm and 355 nm. Thus the main modification of the ChemCam excitation system to enable fluorescence is the addition of three interchangeable frequency multipliers. A flight-qualified mechanical drive train and control box used for the camera filter wheel, for example that used on board the *Beagle 2 Lander*[25,26] could rotate the frequency multipliers into position. Figure 1 is a system level block diagram of the new instrument.The emission collection system has one main modification, the placement of a band pass notch filter in front of each of the three spectrometers. These filters block the excitation wavelengths allowing the natural fluorescence to pass through to the spectrograph. The emission collection system (Figure 2) will employ the Schmidt telescope, the original three ChemCam spectrographs, and the addition of three notch narrow band pass filters (266 nm, 355 nm, and 532 nm). The only non-flight qualified hardware of the entire fluorescence instrument design are the frequency multipliers and the non-moving band pass notch filters as every other system component has been flight qualified. An estimate of these additional components is less than a kilogram of mass and in the tens of thousands of dollars.

### UV fluorescence of organics and minerals relevant to Mars

Many organic molecules, particularly polycyclic aromatic hydrocarbons (PAHs) fluoresce (See Table 1). Polycyclic aromatic hydrocarbons have been found in Martian meteorites, and are the primary form of organic matter found in many meteorites [27]. Biomolecules occurring as amino acids have been detected in the interstellar medium (ISM) [28]. Hence, PAHs and amino acids are plausible candidates for organics on Mars. Storrie-Lombardi et al. [4] detected fluorescence, using a camera and filter wheel, of the three- ring-PAH anthracence, the four ring pyrene, and the five-ring perylene when excited at 365 nm. The fluorescence characteristics of several PAH’s are listed in Table 1. Also listed are the fluorescent characteristics of the aromatic amino acids, phenylalanine, tyrosine, and tryptophan. In addition other biomolecules can be detected and identified using fluorescence as shown for ATP and NADH in Table 1. For a more detailed list of fluorescence of common biomolecules and detection of biomolecules by fluorescence refer to the Additional file 1 which is based on Smith et al. [29].

**Table 1:** Common organics (polycyclic aromatic hydrocarbons, PAH) of extraterrestrial origin (meteorites, interstellar medium (ISM)), and potiential biomolecules that could be on the surface of Mars, excitation and emission wavelength peaks, and reference

**Biomolecules**	**Description**	**Excitation λ**	**Emission λ**	**Reference**
ATP	ubiqitous metabolite	280 nm	420 nm	Katayama et al. [30]
Phenaylanine	amino acid in ISM	260 nm	282 nm	Seaver et al. [31]
NADH	ubiqitous metabolite	350 nm	460 nm	Richards-Kortum et al. [32]
Tyrosine	amino acid in ISM	275 nm	304 nm	Nevin et al. [33]
Tyrptophan	amino acid in ISM	280 nm	353 nm	Alimova et al. [34]
**Chemical organics**				
Benzopyrene	PAH in Mars meteorites	383 nm	435 nm	Kuijt et al. [35]
Chrysene	PAH in Mars meteorites	307 nm	370 nm	Kuijt et al. [35]
Napthalene	PAH in Mars meteorites	275 nm	344 nm	Kuijt et al. [35]
Perylene	PAH in Mars meteorites	280 nm	no peak	Pujari et al. [36]
Perylene		440 nm	472 nm	Wilson et al. [37]
Phenanthrene	PAH in Mars meteorites	280 nm	410 nm	Pujari et al. [36]
Phenanthrene		306 nm	361 nm	Kuijt et al. [35]
Pyrene	PAH in Mars meteorites	347 nm	387 nm	Kuijt et al. [35]
Pyrene		342 nm	376, 396 nm	Wilson et al. [37]

## Methods

To ascertain fluorescence response at the constrained excitation wavelengths (266 nm, 355 nm, and 532 nm), we measured the emission spectra for five PAHs (pyrene, phenanthrene, naphthalene, naphthol, and cresol) individually and when combined with a mineral in a 50/50 mixture by volume. The polycyclic aromatic hydrocarbons, (pyrene, phenanthrene, naphthalene, cresol, and naphthol), powdered dolomite, and Ca perchlorate were purchased from Sigma Aldrich (St. Louis, MO). Powdered dolomite was used as the mineral since it is a magnesium carbonate formed under aqueous conditions and possibly on the Martian surface [23].

Even though the primary task of the fluorescence instrument is to identify organics, the identification of minerals, especially water-associated minerals, would enhance the scientific value without adding to the cost.

To determine the feasibility of identifying minerals using the fluorescence instrument, emission spectra of several rock minerals known to be on the surface of Mars were generated for each of the three excitation wavelengths proposed for this instrument. Additionally emission spectra for rock minerals found at Mars Analog environments on Earth and potentially on Mars were measured. The rock mineral specimens (except for jarosite) were from the Utah State University Geology Department mineralogy and igneous petrology collection. Jarosite was collected from Panoche Valley in California by the research team.

All of the data presented in this paper were taken with a Shimadzu RF-1501 Fluorometer set to a resolution of 5 nm. This instrument has a wavelength accuracy of ±1.5 nm and a signal to noise ratio of 150:1 [38]. Our standard deviation based on triplicate sample measurements is less than 1%, therefore we suggest the inhomogeneity of a sample could introduce a greater error than the that introduced by the method. A custom angled cuvet for powdered mixtures and a sample holder able to hold rocks was positioned in the fluorometer to obtain optimal emission spectra. Each specimen was excited by the fluorometer xenon flash lamp, at 266 nm, 355 nm, and 532 nm and the emission spectra scanned from 220–900 nm, 350–900 nm, and 530–900 nm respectively.

## Results

**Figure 3:**
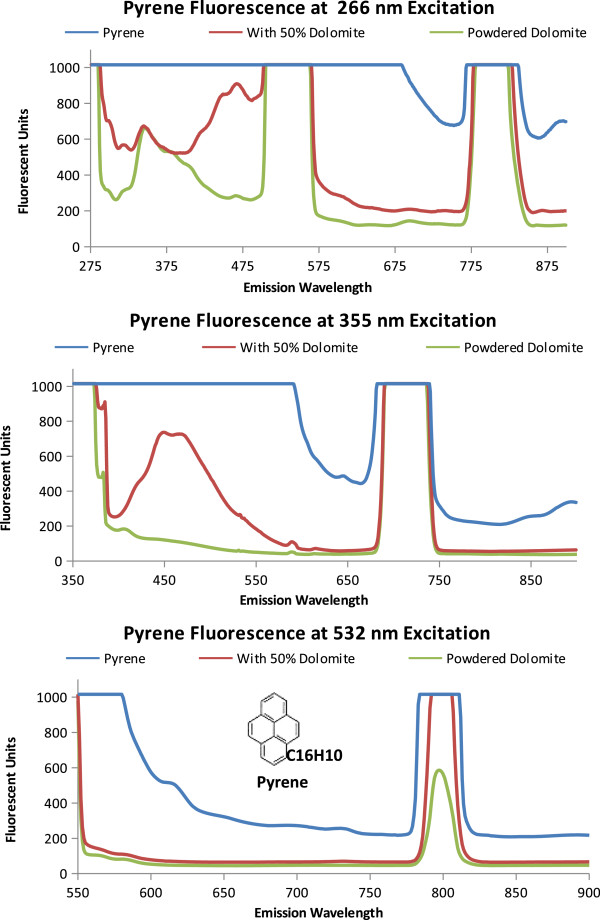
**Pyrene Fluorescence.** Each graph is the emission spectra of pyrene, powdered dolomite, and a 50/50 by volume mixture of dolomite and pyrene when excited at proposed excitation wavelengths (266 nm, 355 nm, and 532 nm). The molecular structure and formula is shown on the 532 nm emission graph.

**Figure 4:**
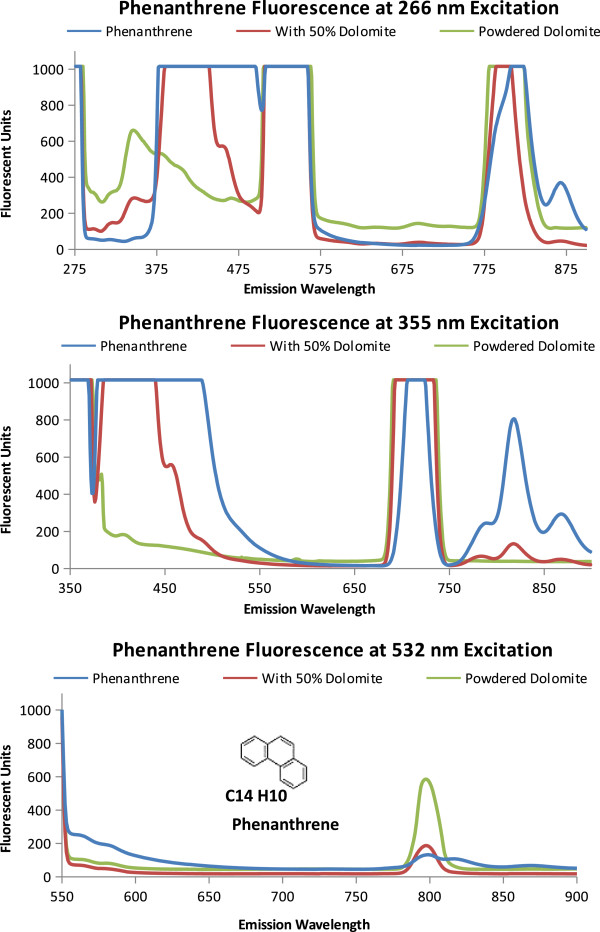
**Phenanthrene Fluorescence.** Emission Spectra of phenanthrene, powdered dolomite, and a 50/50 by volume mixture of dolomite and phenanthrene when excited at proposed excitation wavelengths (266 nm, 355 nm, and 532 nm). The molecular structure and formula is shown on the 532 nm emission graph.

**Figure 5:**
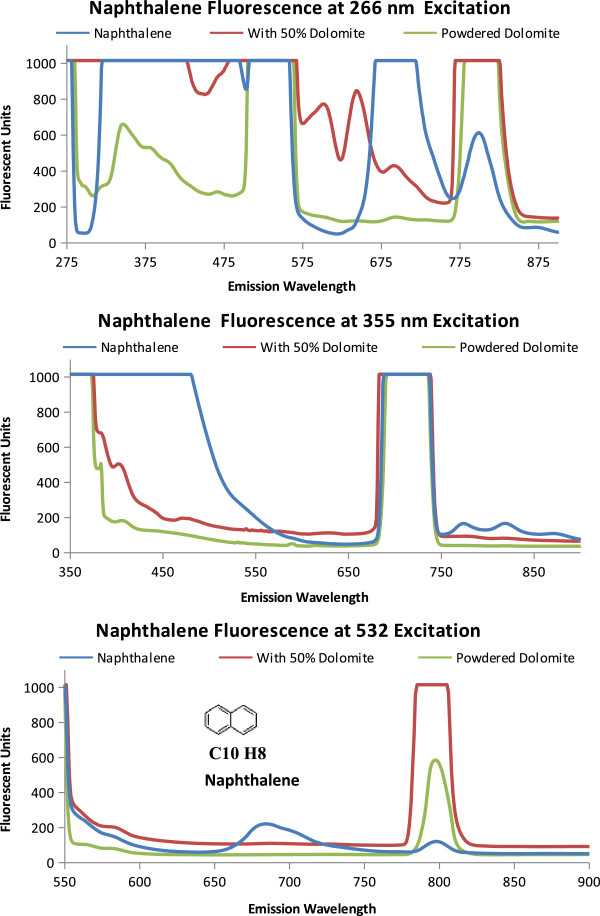
**Naphthalene Fluorescence.** Emission Spectra of naphthalene powdered dolomite, and a 50/50 by volume mixture of dolomite and naphthalene when excited at proposed excitation wavelengths (266 nm, 355 nm, and 532 nm). The molecular structure and formula is shown on the 532 nm emission graph.

Fluorescence and quenching results for three polyaromatic hydrocarbons likely to be on Mars, pyrene, phenanthrene, and naphthalene, are shown in Figures 3, 4, and 5 and described in Table 2. The emission spectra for the individual PAH is compared with a 50% PAH 50% powdered dolomite by volume mixture and the spectrum for powdered dolomite.

The fluorescence from pyrene is shown in Figure 3. As shown in Figure 3A, when excited at 266 nm pyrene saturates the instrument detectors causing a flat line at the highest instrument readings (1000 Fluorescent units). The powdered dolomite and the 50/50 mixture of pyrene and dolomite have an emission peak at 340 nm. This dolomite peak at 340 nm was also seen in the 50/50 mixture of pyrene and dolomite. The addition of the dolomite quenched the pyrene signal by lowering the fluorescent units to 600 for the 50% pyrene, 50% dolomite mixture compared with over 1000 fluorescent units for pyrene alone. Rather than fluorescence, the peaks from 498 to 532 nm, and the peak from 770 to 810 nm are likely due to instrumentation setup and interactions between the excitation wavelength and the samples analyzed (dolomite and pyrene) which produces a resonance frequency at two times the excitation wavelength (2λ, 2 × 266 = 532 nm). Resonance frequencies can also occur at other intervals such as three times the excitation wavelength (3λ =798 nm) as seen in the peak between 770 and 810 nm. As seen in Figure 3B with the 355 nm excitation, the fluorescence emission detectors are saturated until 590 nm where the fluorescence sharply drops to around 500 fluorescent units and continues to reduce. The 710 nm emission peak (2λ) should be regarded as an artifact of the spectrometer grating instead of fluorescence. The 440 nm emission from the 50% pyrene to 50% dolomite mixture by volume both enhances the dolomite peak at 440 nm and reduces the pyrene emission. For pyrene, as the excitation energy decreases, so does the variation in emission spectra (from graphs 3A to 3C). In 3C, when excited at 532 nm, pyrene has a small emission peak at 615 nm. The peak at 790 nm (1.5 λ) frequency resonance is less for dolomite than for pyrene, and the 50% pyrene 50% dolomite by volume mixture.In Figure 4 the three-ringed phenanthrene displays distinguishing emission spectra at each excitation wavelength. In Figure 4A, only phenanthrene has an emission peak at 861 nm (266 nm ex) and fluoresces from 351 to 500 nm. At 355 nm excitation the 50% phenanthrene 50% dolomite by volume mixture and phenanthrene both have an emission peak at 818 nm. When Phenanthrene is excited at 532 nm, the 818 nm peak seen at 355 excitation disappears.Naphthalene, as shown in Figure 5, also has an identifiable emission spectrum as pronounced by the 683 nm emission peak at 532 nm excitation. Naphthalene and Phenanthrene have similar spectrum profiles at 355 nm excitation, the higher peak intensity at 818 nm distinguishes Phenanthrene from Naphthalene.

**Table 2:** Summary of PAH spectrum characteristics for each of the polycyclic aromatic hydrocarbons shown above as measured for this study using a Shimadzu 1501 Fluorometer

**PAH**	**266 nm ex spectrum comparison**		**355 nm ex spectrum comparison**		**532 nm ex spectrum comparison**	
	**Fluorescence(nm)**	**Quenching(nm)**	**Fluorescence (nm)**	**Quenching (nm)**	**Fluorescence (nm)**	**Quenching (nm)**
Pyrene	260 to 650		355 to 590		532 to 590	
Phenanthrene	352 to 500, 861	291 to 351, 501	377 to 507, 818	376	550 to 650	
Naphthalene	315 to 557,	291 to 314	355 to 506, 766, 818		683	
	640 to 740	558 to 640				
Naphthol	321 to 400	291 to 320	410			790
Cresol	380		355 to 450, 770	710		790

**Figure 6:**
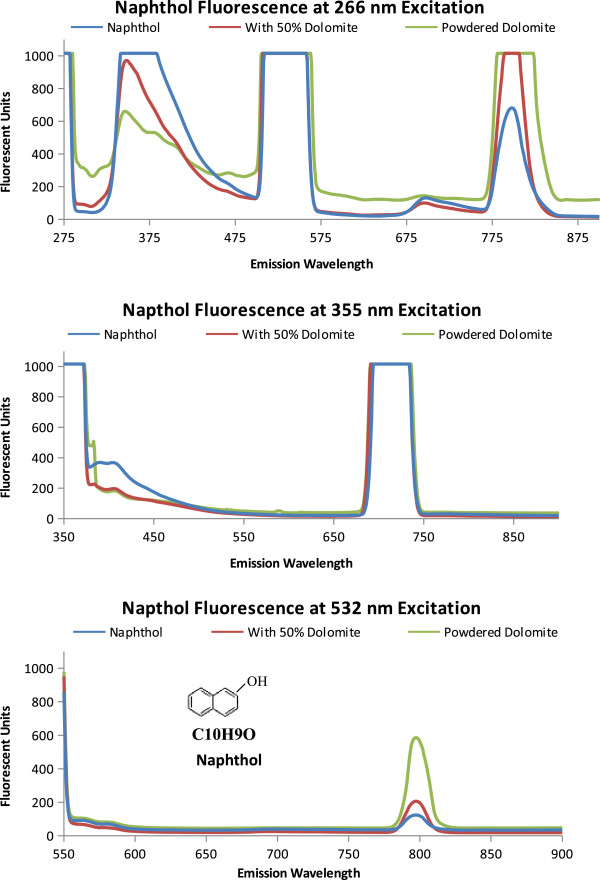
**Napthol Fluorescence.** Emission spectra of napthol, powdered dolomite, and a 50/50 mix when excited excitation wavelengths 266 nm, 355 nm, and 532 nm. The molecular structure and formula is shown on the 532 nm emission graph.

**Figure 7:**
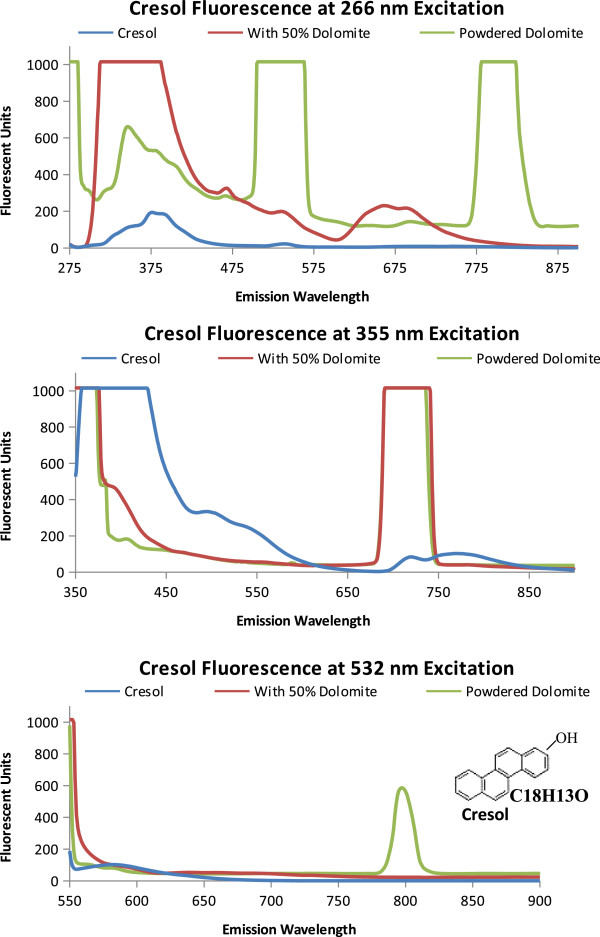
**Cresol Fluorescence.** Emission spectra of cresol, powdered dolomite, and a 50/50 mix when excited excitation wavelengths 266 nm, 355 nm, and 532 nm. The molecular structure and formula is shown on the 532 nm emission graph.

**Figure 8:**
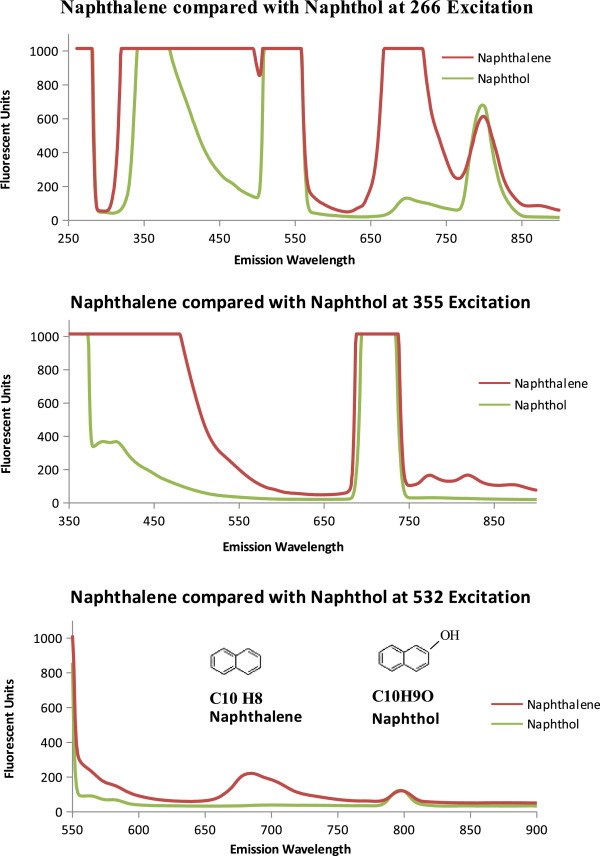
**Naphthalene and Napthol Fluorescence.** Emission spectra of Naphthalene at 266 nm, 355 nm, and 532 nm compared with Napthol to investigate the effect of a hydroxyl group. The molecular structure and formula is shown on the 532 nm emission graph for each molecule.

The Martian surface contains superoxides [39] and therefore, oxidized polycyclic aromatic hydrocarbons (naphthol and cresol) were measured and compared to species without the addition of a hydroxyl group. As shown in Figure 6, naphthol, powdered dolomite, and the 50% naphthol, 50% dolomite by volume mixture have relatively similar emission spectrum. Some differences are the relative fluorescent units at 266 nm excitation and the naphthol emission peak at 355 nm excitation. Cresol, as shown in Figure 7, has a distinct emission spectrum for each excitation wavelength. At 266 nm excitation the cresol spectrum has a less fluorescence than dolomite. At 355 nm Cresol has an enhanced fluorescence followed from 380 to 440 nm. At 532 nm the dolomite has an emission peak centered at 796 nm. The hydroxyl radical alters the emission spectrum of the PAHs, by reducing the fluorescence as seen between the Naphthalene and Naphthol in Figure 8.

**Figure 9:**
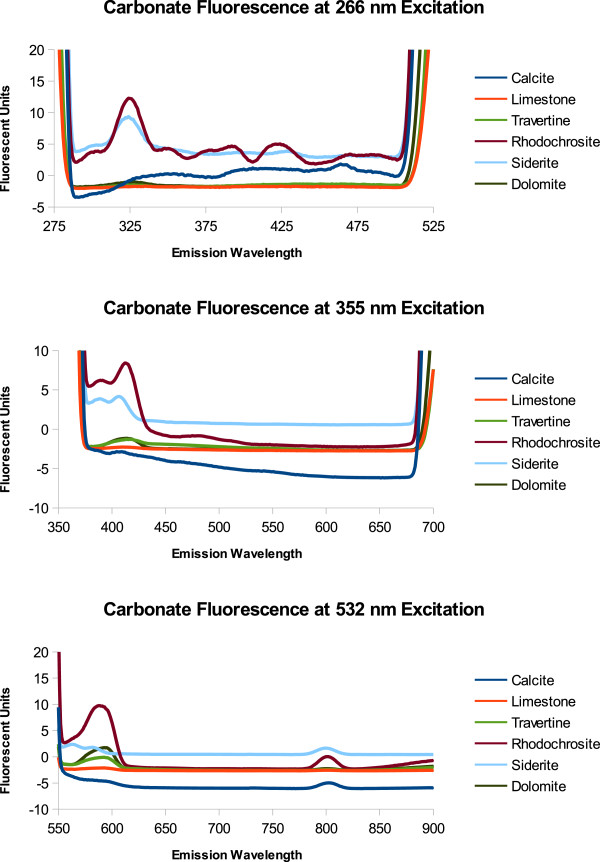
**Carbonate Fluorescence.** The spectrum on the left side of the graph is the tail end of the excitation peak. The rising spectrum on the right side of the graph is the beginning of the 2 λ peak. The 2 λ emission peak is an artifact of the grating within the Shimadzu Fluorometer and is not likely due to mineral fluorescence. Furthermore a 1.5 λ emission peak can be seen for the carbonates. This also is likely due to the instrument conditions rather than mineral fluorescence.

**Figure 10:**
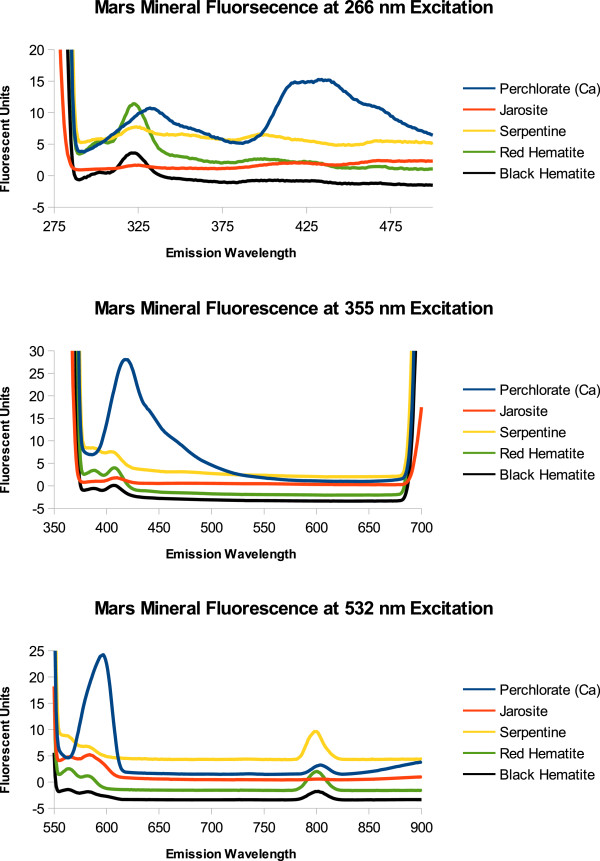
**Fluorescence of Mars Minerals.** The spectrum on the left side of the graph is the tail end of the excitation peak. The rising spectrum on the right side of the graph is the beginning of the 2 λ peak. The 2 λ emission peak is an artifact of the grating within the Shimadzu Fluorometer and is not likely due to mineral fluorescence. Furthermore a 1.5 λ emission peak can be seen for Ca perchlorate. This also is likely due to the instrument conditions rather than mineral fluorescence.

We measured minerals known to be on the surface of Mars. Non-contact or remote mineral identification would improve the scientific merit of the instrument. Also, as reported by Smith et al. [26] the detection of biomolecular fluorescence was influenced by mineral fluorescence. The data in Figure 9 shows the emission spectra of carbonates (calcite, limestone, travertine, rhodochrosite, siderite, and dolomite) and Figure 10 shows some water associated minerals (perchlorate, hematite (red and black), serpentine, and jarosite), known to be on the surface of Mars, when excited at 266 nm, 355 nm, and 532 nm. The portion of the spectra graphed is from the tail end of the excitation peak to the beginning of the 2λ resonance fluorescence peak. Fluorescence beyond the 2λ peak is unexpected due to the large Stokes shift and lower energy output. At 266 nm excitation rhodochrosite, siderite, hematite, and perchlorate have distinct emission peaks. At 355 nm excitation, most minerals have a small fluorescence peak between 400 and 410 nm with the exception of perchlorate which fluoresces about fifty times brighter at 538 nm and 589 nm. At 532 nm the peaks are likely due to scattering of light or a grating effect at 1.5 λ rather than fluorescence, except for the small emission peak at 726 nm from perchlorate.

## Discussion

The survey of PAH and mineral fluorescence performed in this study illustrates that the 1064 nm laser available in the flight ready ChemCam instrument modified with the addition of the three frequency multipliers (266 nm, 355 nm, 532 nm) and band pass filters would provide an excellent site survey tool in near real time based on fluorescence measurements. Using mostly flight qualified hardware reduces the risk and cost associated with new instrumentation. A modified version of the ChemCam instrument described in this paper would be able to recognize biomolecules, PAHs, and minerals. Table 3 lists the material identifiable at the specified wavelengths.

**Table 3:** Biomolecules, organics, and minerals detectable by fluorescence by measuring the emission spectra, when excited at the corresponding wavelength

**266 nm excitation**	**355 nm excitation**	**532 nm excitation**
Adenosine Tri phosphate (ATP), Tryptophan, Tyrosine, Pyrene, Phenanthrene, Naphthalene, Mg Perchlorate, Hematite, Calcite, Siderite	Dipicolinic acid, NADH, Phenanthrene, Naphthol, Cresol, Mg Perchlorate, Siderite, Rhodochrosite, Hematite	Jarosite, Rhodochrosite, Dolomite, Naphthalene

With the operational flexibility of remote measurements, an outcrop could be surveyed a few meters away to avoid risks associated with movement. This greatly reduces the chance of mechanical failure from the rover getting stuck and expands the range of the target selection area. Future research will focus on expansion of the target database to include meteorites and tektites and on taking fluorescent measurements under conditions that are similar to those anticipated on Mars to determine the distances at which fluorescence detection is feasible.

## Conclusions

We have developed a conceptual design for a fluorescence-based instrument for stand-off detection of organics on Mars. Our design is based on the technology developed for the ChemCam LIBS instrument that has been constructed for the Mars Science Laboratory mission. The key design change would be the addition of wavelength multiplexers to create higher harmonics of the 1064 nm laser that is presently on ChemCam. These lower wavelengths allow for fluorescence of both organic and mineral detection. To demonstrate this we have investigated the fluorescence properties at specific harmonic wavelengths of organics and minerals relevant to Mars. From this design work and data collection, our main conclusions are:

1) Fluorescence can be used to detect organics (PAH), and minerals that are expected to be on Mars.

2) The fluorescence instrument proposed is comprised almost entirely of flight-qualified Mars surface operational hardware reducing the risk and cost.

3) Feasibility of the instrument design offers a high scientific return (detection of organics) while expending minimal resources (time, development costs).

4) This instrument should be considered for the next mission to Mars as a site survey tool.

## Competing interests

The authors declare that they have no competing interests.

## Authors’ contributions

HDS prepared the samples and carried out the fluorescence measurements, participated in discussions of the instrument design, and drafted the manuscript. CPM advised on all aspects of the ChemCam Instrument, participated in data analysis and discussion, and drafted a manuscript section. AGD drafted two figures for the manuscript, participated in fluorescence measurements and instrument design discussion. RCS advised on the all aspects of this research, assisted in data analysis and discussions. AJA advised on and provided the supplies for the PAH, and participated in data interpretation and discussions. PRG advised on the mineralogy aspects of this research and participated in data interpretation and discussions. All authors read and approved the final manuscript.

## Supplementary Material

Additional file 1: Table S1 Fluorometer Mineral List. Rocks analyzed using a custom rock holder in a Shimadzu 1501 fluorometer excited at 266 nm, 355 nm, and 532 nm. **Figure S1.** Varied Silica Content Emission Spectra. Igneous Rocks classified by silica content. Basalt has the lowest silica content, while Dacite has the highest. The middle silica content (Andesite) has the highest fluorescence. **Figure S2.** Sulfates Emission Spectra. Elemental sulfur compared with sulfate compounds. **Figure S3.** Oxides Emission Spectra. Ilmenite an TiO abundant at impact sites and on the Moon, compared with Pyrolusite a mineral that branches similar to trees, and Magnetite a known Mars mineral. **Figure S4.** Same Composition Emission Spectra. Fluorescence from three minerals with the same composition, but formed under different conditions. Click here for file
