# Supplementary material for: An instrument design for non-contact detection of biomolecules and minerals on Mars using fluorescence
Source: J Biol Eng. 2014 Jul 1;8:16. doi: 10.1186/1754-1611-8-16 (PMC4107600; doi:10.1186/1754-1611-8-16)
Supplement: Additional file 1: Table S1 — Fluorometer Mineral List. Rocks analyzed using a custom rock holder in a Shimadzu 1501 fluorometer excited at 266 nm, 355 nm, and 532 nm. Figure S1. Varied Silica Content Emission Spectra. Igneous Rocks classified by silica content. Basalt has the lowest silica content, while Dacite has the highest. The middle silica content (Andesite) has the highest fluorescence. Figure S2. Sulfates Emission Spectra. Elemental sulfur compared with sulfate compounds. Figure S3. Oxides Emission Spectra. Ilmenite an TiO abundant at impact sites and on the Moon, compared with Pyrolusite a mineral that branches similar to trees, and Magnetite a known Mars mineral. Figure S4. Same Composition Emission Spectra. Fluorescence from three minerals with the same composition, but formed under different conditions. [file 1754-1611-8-16-S1.docx]

**Additional file 1**

**Supplemental Information for** *An Instrument Design for Detecting Biomolecules and Minerals on Mars using Fluorescence.* Submitted to JBE

Fluorescence measurements were taken on a suite of minerals in addition to those reported in the this manuascript. Table S1 lists the minerals by name, the mineral class, and the rock color. Figures S1 to S5 compare some of these minerals and their fluorescence properties.

Table S1 Fluorometer Mineral List. Rocks analyzed using a custom rock holder in a Shimadzu 1501 fluorometer excited at 266 nm, 355 nm, and 532 nm.

| **Mineral** | **Class** | **Rock Color** |
| --- | --- | --- |
| Albite | Plagioclase(Na) | White |
| Andesite | Ingneous (52-63%) | Black and Gray |
| Apatite | Phosphate (Ca) | green |
| Augite | Pyroxene(Ca-Na) | black and green |
| Basalt | Ingneous (<52%) | Black |
| Biotite | Phylosilicate (K) | Black Mica |
| Calcite | Carbonate (Ca) | Clear |
| Dacite | Ingneous (>63%) | gray and white |
| Dolomite (Crystal) | Carbonate+ Mg | white Crystals |
| Dolomite (Green) | Carbonate +Mg | Green planar |
| Dolomite (White) | Carbonate+ Mg | white planar |
| Enstatite | Silicate (Mg) | Green |
| Fluorite | Halide (CaF) | Clear |
| Garnet | Silicate | Deep Red |
| Gypsum (Clear) | Halide/Sulfate | Clear |
| Halite | Halide (NaCl) | Clear |
| Hematite (black) | Oxide (FeO_3_) | Black |
| Hematite (red) | Oxide (FeO3) | Red more Fe |
| Hornblend | Amphibole/ silicate | Black |
| Imenite | Oxide (FeTiO_3_) | Black |
| Jasper | Silicate | Red |
| Jarosite | Sulfate (KFe3(SO_4_)_2_(OH)_6_ | Yellow, Brown,Red |
| Limestone | Carbonate (Ca) | White |
| Magnetite | Oxide (FeO_4_) | Black |
| Microcline | Feldspar (K) | White |
| Muscovite | Phylosilicate (K, Al) | Clear Mica |
| Olivine | Silicate(Fe) | Green |
| Perchlorates | Oxide (XClO_4_) | White grains |
| Phlogopite | Phylosilicate (K Mg) | Brown Mica |
| Pyrolusite | Oxide (MnO_2_) | Black Branching pattern |
| Quartz(clear) | Silicate | Clear |
| Quartz (smoky) | Silicate(K, Mg,Fe) | Gray |
| Rhodochrosite | Carbonate (Mn) | Pink |
| Serpentine | Phylosilicate (Mg) | Green, Reddish intrusions |
| Siderite | Carbonate (Fe) | brown |
| Sulfur | Sulfate (S) | Bright Yellow |
| Titanite (Spene) | Silicate(CaTiO)SiO_4_) | Black |
| Travertine | Carbonate (Ca) | Milky white to brown |

Figure S1 Varied Silica Content Emission Spectra. Igneous Rocks classified by silica content. Basalt has the lowest silica content, while Dacite has the highest. The middle silica content (Andesite) has the highest fluorescence.

Figure S2 Sulfates Emission Spectra. Elemental sulfur compared with sulfate compounds.


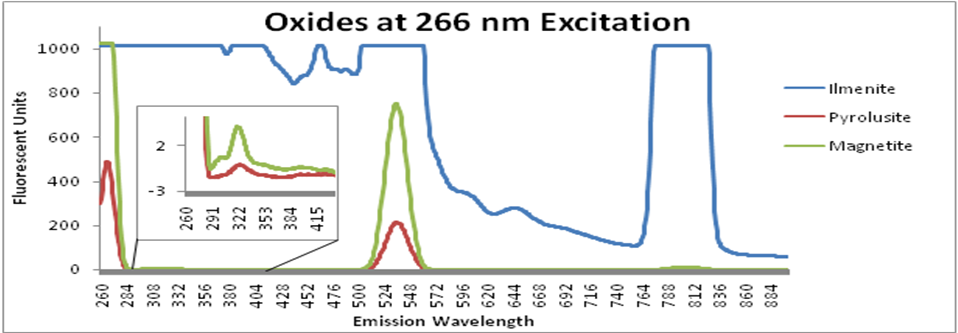


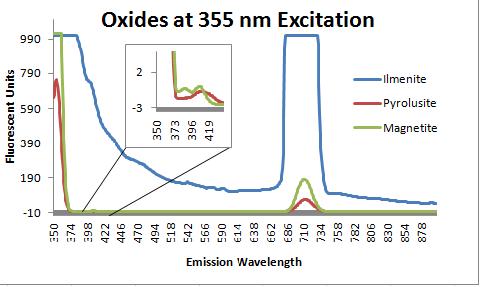


Figure S3 Oxides Emission Spectra. Ilmenite an TiO abundant at impact sites and on the Moon, compared with Pyrolusite a mineral that branches similar to trees, and Magnetite a known Mars mineral.

Figure S4 Same Composition Emission Spectra. Fluorescence from three minerals with the same composition, but formed under different conditions.
